# Candidate historical events for the emergence of Human Coronavirus OC43: A critical reassessment of the molecular evidence

**DOI:** 10.1371/journal.pone.0285481

**Published:** 2023-05-08

**Authors:** Brandon Shaw, Derek Gatherer

**Affiliations:** Division of Biomedical & Life Sciences, Faculty of Health & Medicine, Lancaster University, Lancaster, United Kingdom; University of Malaga: Universidad de Malaga, SPAIN

## Abstract

The “Russian Influenza”-coronavirus theory (RICT) proposes that the pandemic of 1889–1892, conventionally regarded as an influenza pandemic, was caused by the emergence of human coronavirus OC43 (HCoV-OC43) as a zoonosis of bovine coronavirus (BCoV). RICT is based on a Bayesian phylogenetic calculation of the date of the most recent common ancestor (MRCA) of HCoV-OC43 and BCoV. The theory also draws on comparison of both symptoms and some epidemiological parameters of the best studied coronavirus pandemic, i.e. COVID-19, with those reported in 1889–1892. The case is completed with circumstantial evidence involving a panzoonotic among cattle in the decade prior to the “Russian Influenza”, with characteristics suggesting it may have been caused by BCoV. In this paper, we review the Bayesian phylogenetic evidence for RICT, replicating previous studies and adding our own, in each case critically reviewing the suitability of the datasets used and the parameters applied. We conclude that the most probable date for the MRCA of HCoV-OC43 and BCoV is 1898–1902. This is a decade too late for compatibility with RICT but happens to coincide with another serious outbreak of respiratory illness, reported in both the USA and the UK, in the winter of 1899–1900.

## Introduction

On 31^st^ May 2020, while the world was enduring the first wave of the COVID-19 pandemic, an article appeared in the UK Sunday newspaper *The Observer*, that reignited an almost forgotten fifteen year old debate about the origins of the “Russian influenza” pandemic of 1889 to 1892. That article was “Did a coronavirus cause the pandemic that killed Queen Victoria’s heir?” [1]. The heir in question was Prince Albert Victor, Duke of Clarence and Avondale, the eldest son of the Prince of Wales and therefore at that time second in line to the British throne. The 28 year old Duke died on 14^th^ January 1892 from “a severe attack of influenza, accompanied by pneumonia”, as *The Times* had described it three days previously. Such tragedies were not uncommon at the time. In early 1892, the UK was just entering its third year of a pandemic that *The Times* had first referred to as “the Russian influenza” on 6^th^ January 1890.

In 1894, H. Franklin Parsons wrote a review setting the events of the preceding few years in the context of what appeared to be consistently declining mortality from influenza in England and Wales over the four decades since the previous pandemic influenza event of 1847–1848 [2]. In a later paper [3], Parsons remarked that influenza “had subsided almost to vanishing point” prior to 1890. Annual mortality officially attributed to influenza had decreased from 460 deaths per million (dpm) in the pandemic year of 1848 to less than 10 dpm per annum average for the period 1874 to 1889. Such a 10 dpm annual figure for seasonal influenza is low even by modern standards. For instance, the bad winter seasonal H3N2 strain of 2017–2018 (dubbed “Aussie flu” by the British media, in a tradition going back through the “Spanish influenza” of 1918 to the “Russian influenza”) produced 253 dpm [4]. But Parsons calculated cumulative influenza mortality for years 1890–1892 inclusive, at what must have been a horrifying 1270 dpm, more than 40-fold greater than what would then have been regarded as a typical cumulative seasonal influenza death toll for any three year period.

Even this may have been an underestimate. Cumulative excess deaths for England and Wales for the same three years, over the average of the preceding five (1885–1889 inclusive), were 2275 dpm. To put this into a modern context, excess deaths for the same two nations within the UK in 2020–2021 inclusive, compared to the preceding five years (2015–2019 inclusive), totalled 1866 dpm. Even if the previously mentioned “Aussie flu” winter is removed from the background (using instead only 2015–2017 inclusive and 2019), the excess deaths figure for the COVID-19 years of 2020–2021 inclusive, still only reaches 1916 dpm (Table 1). Although COVID-19’s excess deaths total will probably be higher still once 2022 is included in the calculation, the seriousness of the pandemic known as “the Russian influenza” is undoubted [5]. The shock it produced was all the greater due to the prolonged period of very low seasonal mortality from influenza that had preceded it [2, 3].

**Table 1:** Excess mortality for England & Wales calculated for the “Russian influenza” pandemic (1890–1892 in the UK) and COVID-19 (2020 and 2021 only).

Year	Deaths per million (dpm) *per annum* (*p*.*a*)	Excess dpm *p*.*a*. (in brackets excluding 2018 from background)	Pandemic event total excess dpm (excluding 2018)
**2021**	9811	749 (773)	1866 (1916)
**2020**	10180	1118 (1142)	
**2019**	8931		
**2018**	9161 (“Aussie flu” year)		
**2017**	9077		
**2016**	8993		
**2015**	9150		
**………**			
**1892**	19023	187	2275
**1891**	20213	1377	
**1890**	19547	711	
**1889**	18221		
**1888**	18161		
**1887**	19073		
**1886**	19521		
**1885**	19204		

These are raw deaths, so do not take into account the differing age structures of the population in the two periods compared. It may be noted that dpm was generally about twice as high in the late 19^th^ century as today. Figures for 2022 were not available at time of writing. 1890–1892 were the years when the UK suffered the “Russian influenza”, but the global pandemic commenced in 1889 and was still affecting some countries as late as 1894. Excess dpm *p*.*a*. are calculated against the average dpm *p*.*a*. of the 5 years pre-pandemic. For COVID-19, excess dpm *p*.*a*. is also calculated against 2019 and 2015–2017 only, to exclude the effect of “Aussie flu” (alternative total given in brackets). Source: kindly provided on email request from Office for National Statistics, UK.

There was little doubt at the time concerning the aetiology of the pandemic. Despite the relative scarcity of influenza cases in the mid-19^th^ century (at least if Parsons’ figures are to be believed), descriptions by Victorian doctors of influenza [e.g. 6, 7] show that they were dealing with the same set of symptoms that would be recognisable as that disease today. The practitioners of late Victorian Britain had no recourse to laboratory testing, and could only diagnose on the basis of signs and symptoms, but in the early 1890s they were nevertheless still experienced enough to be confident that they had an influenza pandemic on their hands–the first since the late 1840s.

Doubts about this standard account began to arise in 2005 when Vijgen et al. [8] produced the first complete genome of human coronavirus OC43 (HCoV-OC43: family *Coronaviridae*; sub-family *Orthocoronavirinae*; genus *Betacoronavirus*; sub-genus *Embecovirus*; species name *Betacoronavirus 1*). The first decade of this millennium was a time when Bayesian phylogenetic approaches were beginning to be used extensively within molecular virology and the new HCoV-OC43 genome was analysed using early versions of both BEAST [9] and TempEst [then called Path-O-Gen: 10]. Only that one HCoV-OC43 genome was available, but several long collection-dated sequences of its close relative bovine coronavirus (BCoV: same taxonomic hierarchy as above) were already in GenBank. The date of their most recent common ancestor (MRCA) was estimated at 1873, using Path-O-Gen with a maximum likelihood phylogenetic tree as input. On application of BEAST, the corresponding figures were 1890 (coalescent model, presumably constant size, although the paper is not specific) and 1893 (exponential growth model). Furthermore, HCoV-OC43 was distinguished from BCoV by a deletion not shared by any other betacoronavirus species, which the principle of parsimony would suggest made it more likely that the common ancestor was undeleted–and hence more likely to have had a bovine host. HCoV-OC43 was therefore postulated to have emerged as a zoonosis from cattle, rather than BCoV having emerged by the reverse anthroponosis.

The predicted dates of the MRCA were not the only suggestive factor. Vijgen et al. [8] also drew attention to the 1870–1890 period of mass culling of cattle due to an outbreak of infectious respiratory disease, which would be precisely the kind of circumstance that would be conducive to zoonosis, as farm and slaughterhouse staff worked their way through the disposal of large cohorts of diseased animals. The coronavirus hypothesis also helped to explain another peculiarity of “the Russian influenza”, that it involved frequent neurological complications. This is not typical of influenza [although not unknown, e.g. 11] but has been reported for HCoV-OC43 patients [12, 13]. Similarly, COVID-19’s famous effects on taste and smell are mediated by the neurotropism of SARS-CoV2 [14].

Vijgen et al. subsequently [15] analysed two further datasets of BCoV and HCoV-OC43 sequences, producing MRCA dates for the spike gene (1902) and nucleocapsid gene (1910). The later MRCA dates for these subsequent datasets possibly contributed to the disappearance of RICT for the next 15 years. Nevertheless, McKie’s topical *Observer* article brought the original paper of Vijgen et al. [8] back to the attention of the virology research community, and new studies followed over the next two years.

Brussow and Brussow [16], Berche [17] and Erkoreka et al. [18] looked at various clinical and epidemiological aspects of “Russian influenza”, concluding with varying degrees of certainty that these bear far more resemblance to those expected of a coronavirus than an influenza virus. Meanwhile, Bidokhti et al. [19] and subsequently Forni et al. [20] had updated the molecular phylogenetic analysis to incorporate the genome sequences of the many strains of both HCoV-OC43 and BCoV that have become available since the work of Vijgen et al. [8]. Bidokhti et al. [19] placed the MRCA at 1899 and Forni et al. [20] at 1923. Otieno et al. [21] produced 5 datasets giving MRCAs around 1925 (from visual assessment of their phylogenetic trees, exact dates are not specified in their paper). Most importantly, Otieno et al. [21] also cast doubt on BCoV as the zoonotic source of HCoV-OC43. These previous MRCA dating studies are summarised in Table 2. As of late 2022, the status of the “Russian influenza”-coronavirus theory (RICT) appears to be that clinical and epidemiological studies have argued in its favour, whereas molecular studies have tended to produce MRCA dates that are slightly too late.

**Table 2:** Published studies calculating date of MRCA of BCoV and HCoV-OC43.

Authors	Gene(s): Product(s)	Method	MRCA (95% HPD)
Vijgen et al. [8]	S: spike	Path-O-Gen (ML tree)	1873 (1815–1899)
		BEAST (coalescent)	1890 (1859–1912)
		BEAST (exponential growth)	1893 (1866–1918)
Vijgen et al. [15]	S: spike	BEAST 1.2 (relaxed exponential clock, HKY+G, coalescent constant size)	1902 (1802–1956)
	N: nucleocapsid		1910 (1812–1961)
Bidokhti et al. [19]	S: spike	BEAST 1.6.2 (strict clock, HKY+G+I)	1899 (1884–1915)
Forni et al. [20]	O: ORF1a non-recombinant region	BEAST 1.10.4 (relaxed lognormal clock, coalescent constant size)	1923 (1872–1967)
Otieno et al. [21]	W: whole genome, S: spike, N: nucleocapsid, M: membrane proteins	BEAST 1.10.4 (relaxed clock, HKY+G+I, Bayesian skyline)	Trees shown, but no specific dates given

The “Method” column shows the clock, substitution and tree models with level of detail given in the article concerned. It may be noted that only the original study of Vijgen et al. [8] gives mean MRCA dates prior to the “Russian influenza”, although all studies encompass the years 1884–1889 within their 95% confidence limits (highest posterior densities–HPDs).

It should also be acknowledged that the long accepted theory, that the 1889–1892 pandemic was indeed caused by influenza, also has its own evidential basis, summarised by Dowdle [22] and updated by Worobey et al. [23]. Much of this relies on seroarchaeology–the detection of antibodies against influenza in historically donated sera. Worobey et al. [23] concluded that influenza type A subtype H3N8 was the cause of the “Russian influenza”. Exposure of the population to this antigenic combination in the 1890s also helps to explain the higher excess mortality from 1918’s H1N1 pandemic among those born in the late 1880s. Their principal exposure to influenza, especially during the crucial early childhood period, would have been to H3N8, giving them poor protection on the arrival of H1N1 in 1918, but conversely greater protection, and therefore lower mortality, when exposed to 1968’s H3N2 “Hong Kong flu” pandemic, as indeed proved to be the case. A related discipline to seroarchaeology is palaeoserology–the detection of antibodies in the dental pulp of cadavers. Ramassy et al. [24] have detected palaeoserological evidence of coronavirus exposure in individuals born between 1864 and 1894, but it is not possible to identify the exact virus to which they were exposed, nor the date of their exposure. The two remaining studies necessary to complete the quadrant—seroarchaeology for coronaviruses, and palaeoserology for influenza–have not so far been performed.

## Methods

We downloaded, constructed or recreated several data sets, as described in Table 3.

**Table 3:** Datasets used in Bayesian phylogenetic analysis.

Name	Original Publication	Provided/ recreated/ constructed	Gene(s): Product(s)	*n* total	*n* OC43	*n* BCoV	*n* others	L
V-2005-S	Vijgen et al. [8]	Recreated	S: spike	16	1	15	0	4119
V-2006-S	Vijgen et al. [15]			17	8	9	0	4131
V-2006-N			N: nucleocapsid	10	3	7	0	1344
B-2013-S	Bidokhti et al. [19]		S: spike	75	4	67	4	4146
F-2022-O	Forni et al. [20]		O: ORF1a	168	167	1	0	12692
O-2022-S	Otieno et al. [21]	Provided (Dataset “D5”)	S: spike	121	21	26	74	4324
O-2022-N			N: nucleocapsid	155	36	31	88	1411
O-2022-M			M: membrane protein	226	51	31	144	696
S&G-2022-O	This paper	Constructed	O: ORF1a	279	186	93	0	12692
S&G-2022-W			W: whole genome					31303

“Provided” data was downloaded from the supplementary files of Otieno et al. [21]. All other datasets were recreated as far as possible by following the specifications in the papers, or constructed for this study (two S&G-2022 sets). *L*: length of alignment in bases. *n*: number of sequences.

Sequence alignments were performed in MEGA using the codon alignment option, where appropriate, of ClustalW and checked by eye for correct frame. Alignments were trimmed according to the boundaries given in each paper where results are being reproduced. Larger sequence sets were first aligned in MAFFT, before re-examination in MEGA. BEAST 1.10.4 [25] was used for Bayesian estimation of the MRCA of OC43 and BCoV. Where possible, we attempted to run the datasets under the same models as given in the original papers and also to vary the models, guided by the philosophy expressed in Drummond and Bouckaert [26, p.145] that “it is more important to identify which aspects of the modelling have an impact on the *answer you care about* than to find the right model” (italics in original).

For dataset F-2022-O, we deduce from Forni et al. [20—Figure 1 of their paper], that the non-recombinant region they identify comprises ORF1a starting at a position about 14% along the gene’s length and ending at 70% along the gene’s length. Since ORF1ab covers positions 210 to 21493 of the reference genome, based on this estimate we construct an alignment of positions corresponding to 2833 to 15524 on the reference genome.

## Results

### Datasets with predominantly HCoV-OC43 and BCoV sequence content

#### Datasets rejected on basis of outgroup problem

We begin by setting aside two datasets, V-2005-S and F-2022-O, although our reconstructed versions of these are provided in our supplementary data for those who wish to explore them further. Our rationale for neglecting these is that they have single BCoV or HCoV-OC43 taxa as outgroups. In V-2005-S all but one of the 16 sequences used are from BCoV. Conversely, F-2022-O has only one BCoV sequence out of 168. Use of sequence sets with such outgroups (whether introduced deliberately, or inadvertently as a result of uneven sequence sampling) are discouraged for Bayesian analysis because sampling of priors is then uneven across the tree [26, p.98].

#### Datasets rejected on the basis of too much branch rate heterogeneity

Having rejected V-2005-S and F-2022-O, we commence analysis of the remaining datasets with application of a relaxed lognormal clock model to each, using, where appropriate, the other model parameters in the original paper from which the dataset is devised. Table 4 summarises these preliminary runs.

**Table 4:** Preliminary Bayesian analysis using a relaxed lognormal clock with the coefficientOfVariation and its upper and lower 95% HPD bounds given.

**Name**	**Substitution model**	**Population model**	**coefficientOfVariation mean**	**Upper bound 95% HPD**	**Lower bound 95% HPD**
V-2006-S	HKY+G	Coalescent constant size	0.13	0.319	< 0
V-2006-N			1.19	2.47	0.211
B-2013-S (75)	HKY+G+I		0.671	0.879	0.486
B-2013-S (71)			0.685	0.894	0.497
S&G-2022-O			0.857	1.03	0.681
	TN93+G+I		0.962	1.16	0.738
			0.972	1.17	0.782
S&G-2022-W	GTR+G+I		1.032	1.24	0.843
		Bayesian skyline	0.980	1.15	0.795

Those datasets with a mean coefficientOfVariation greater than 1, are rejected for further analysis (V-2006-N). Those with 95% HPD overlapping zero are analysed with the strict clock only (V-2006-S).

After this preliminary analysis, we also made the decision to set aside dataset V-2006-N, since the posterior values of the coefficientOfVariation parameter (mean 1.19, 95% HPD 0.211–2.47) suggest that V-2006-N has too much branch rate heterogeneity (mean >1) to be considered reliable [26, p.151]. For S&W-2022-W, the effect is borderline, with mean coefficientOfVariation dipping below 1 when the Bayesian skyline model is used. S&W-2022-W is therefore retained, but only run under a Bayesian skyline model.

#### MRCAs for accepted datasets

Table 5 shows the MRCA dates obtained for those datasets with a mean coefficientOfVariation parameter less than 1, thus indicating an acceptable level of branch rate heterogeneity for application of a relaxed molecular clock. For S&G-2022-W, the mean coefficientOfVariation could be kept below 1, when a Bayesian skyline tree model was employed, but not a coalescent constant size. For all datasets and all runs, the period 1880–1889 –the decade prior to the Russian influenza—lies within the 95% HPD boundaries of the MRCA. However, mean values for MRCAs are all later than 1896, with the exception of V-2006-S. This dataset was run under a strict model as its coefficientOfVariation 95% HPD overlapped zero. As well as this single run under a strict clock giving an earlier MRCA, it is noticeable from Table 5 that, for those datasets analysed using two variants of the relaxed clock model—S&G-2022-W and B-2013-S(71)–the relaxed exponential clock model widens the 95% HPD and brings the mean MRCA forward in time by 6 to 9 years. For the dataset analysed using three different substitution models–S&G-2022-O–the choice of substitution model makes little difference.

**Table 5:** Bayesian phylogenetic analysis of datasets passing coefficientOfVariation parameter test shown in Table 4.

**Dataset**	**Clock**	**Substitution**	**Tree**	**MRCA (95% HPD)**
V-2006-S	Strict	HKY+G	Coalescent constant size	1879 (1847-1907)
B-2013-S (75)	Relaxed lognormal	HKY+G+I		1909 (1869-1942)
B-2013-S (71)				1896 (1840-1940)
	Relaxed exponential			1902 (1835-1949)
S&G-2022-O	Relaxed lognormal			1900 (1835-1953)
		TN93+G+I		1902 (1839-1951)
		GTR+G+I		1898 (1839-1945)
S&G-2022-W			Bayesian skyline	1913 (1880-1946)
	Relaxed exponential			1924 (1874-1958)

B-2013-S was analysed in its original 75 taxon form, then again once the four non-BCoV, non-HCoV-OC43 sequences had been removed.

Table 5 also shows that the mean MRCA for OC43-HCoV and BCoV moves back in time from 1909 to 1896 when the four porcine coronavirus (PHEV) and equine coronavirus (ECoV) sequences are removed, i.e. B-2013-S(75) versus B-2013-S(71). However, this difference is reduced when a relaxed exponential model is applied. Also the use of the non-recombinant region of the genome versus the entire genome–S&G-2022-O versus S&G-2022-W–also draws the mean MRCA value back in time from 1913 to 1898. However, these two runs are not so directly comparable, as the failure of the coefficientOfVariation parameter test (Table 4) for S&G-2022-W under the coalescent constant size model means that the Bayesian skyline is used instead.

*Dataset with extensive non-HCoV-OC43 and non-BCoV sequence content*. O-2022-“D5” was not originally created for the purposes of elucidating the MRCA of BCoV and OC43, and contains several other species of virus within it. Otieno et al. [21] find that HCoV-OC43 and BCoV do not resolve as a single taxon for all three alignments. Otieno et al. [21] present no specific MRCA dates for HCoV-OC43 and BCoV, indeed they question the basic assumption that HCoV-OC43 and BCoV are sister taxa. Table 6 summarizes the tree topologies obtained in the original paper of Otieno et al. [21] and in our three Bayesian phylogenetic trees generated from O-2022-“D5”.

**Table 6:** Comparison of sister taxa obtained for HCoV-OC43 in the original paper of Otieno et al. and in our replication.

Source	Gene	Sister taxon BCoV (% Markov jumps) [posterior]
Otieno et al.	spike	Bovine (35) Murine (19) Camel (14) Rabbit (14) Canine (8) Equine (5)
	nucleocapsid	Porcine (25) Bovine (23) Murine (18) Rabbit (15) Equine (10)
	membrane protein	Camel (75) Murine (6) Rabbit (3) Porcine (3) Equine (2) Bovine (2) Bat (2)
O-2022-“D5”	spike	clade containing BCoV and canine respiratory Cr-CoV [0.999]
	nucleocapsid	clade containing BCoV, PHEV and Cr-CoV [1.000]
	membrane protein	clade containing BCoV, dromedary DcCoV-HKU23 and Cr-CoV [0.597]

Both studies used BEAST 1.10.4 with HKY+G+I as the substitution model and Bayesian skyline as the tree model. Otieno et al. [21] simply specify a relaxed clock, so we used relaxed lognormal. Otieno et al. [21] quantified the probability of the sister taxon using Markov jumps (given in round brackets as %) whereas we used the top posterior probability from the consensus Bayesian tree [given in square brackets]. “Porcine” in Otieno et al. [21] is equivalent to PHEV, “Canine” to CrCoV. “Camel” to DCCoV-HKU-23.

## Discussion

The issue of the origins of the endemic/seasonal human coronaviruses is important because these viruses may provide a model for the future trajectory of SARS-CoV2. Estimation of the MRCA dates of endemic/seasonal human coronaviruses and their closest relatives, may provide us with an estimation of how frequently we expect coronavirus pandemics to occur. For instance, in the case of influenza A, we have solid molecular evidence of four pandemics in the period 1918 to 2009 inclusive, suggesting an average frequency of one every 30 years. For coronaviruses, we have no equivalent information on which to base any estimate. However, if we conclude that the “Russian influenza” was caused by the emergence of HCoV-OC43 by zoonosis from BCoV, then we have two coronavirus pandemics from 1890 to 2020 inclusive, giving an average frequency of one every 130 years. On the other hand, if we regard the SARS-CoV1 event in 2003 as an abortive pandemic, then the average frequency increases to one every 65 years. One might be tempted to argue that the mere 17 years between the appearance of the two variants of SARS-CoV and the mere 11 years between the 1957 and 1968 influenza pandemics, reflects some ecological mechanism at work which cannot be mere chance. However, in the absence of any notion of what that mechanism might be, we must fall back on a theory of stochastic occurrence at a certain background frequency with occasional clusters emerging at random.

Increased knowledge of the origins of HCoV-OC43 therefore provides an opportunity to achieve a greater appreciation of both the long-term risk of another coronavirus pandemic and the potential long-term behaviour of SARS-CoV2, the most recent virus to cause such a pandemic. From the data presented here, we conclude that the MRCA dates of previous studies are largely robust to replication, even though–with the exception of the work of Otieno et al. [21]—we must in all cases recreate the dataset from the descriptions in the original papers. The only study to provide a mean MRCA for HCoV-OC43 and BCoV that is compatible with RICT is that of Vijgen et al. [15] and that this result is reproduced in our dataset V-2006-S. However, this is the only dataset to be compatible with a strict molecular clock. For the remainder, using relaxed clock models, which the Bayesian analysis indicates are more appropriate, the mean MRCA dates range from 1896 to 1924.

Guided, as we are, by the philosophy expressed in Drummond and Bouckaert [26, p.145] that “it is more important to identify which aspects of the modelling have an impact on the *answer you care about* than to find the right model” (italics in original), we proceed to explore those parameters that affect the mean MRCA. It is evident that choice of substitution model is mostly immaterial. When choosing a relaxed clock model, exponential distributions tend to slightly later MRCA dates than lognormal ones. Aside from the issue of relaxed versus strict clock, other commonly varied model parameters appear to make little difference to the issue of RICT.

The above assumes, however, that HCoV-OC43 and BCoV are amenable to Bayesian phylogenetic analysis where a tree-like topology generated by the software is a true representation of the evolutionary history of these viruses. The possible presence of recombination presents some grounds for rejection of that assumption. Forni et al. [20] used 3SEQ to map likely recombination breakpoints within HCoV-OC43. It should be noted that their analysis contained only one BCoV sequence and they did not consider the possibility of recombination between BCoV and HCoV-OC43 subsequent to their hypothesized divergence. Forni et al. [20] identified a non-recombinant region of OC43 which they present diagrammatically in their paper, and which we attempted to replicate in our dataset F-2022-O. Since this contained only one sequence of BCoV, however, we rejected it on the basis of the outgroup issue [26, p.98], although we applied their recommended non-recombinant region in our own dataset S&G-2022-O.

An even stronger objection to the tree-like topology assumption for the evolution of BCoV and HCoV-OC43, comes from Otieno et al. [21], who posited extensive recombination between several members of the genus *Betacoronavirus*. Our replication of their Bayesian consensus trees shows that both canine respiratory coronavirus (Cr-CoV), porcine coronavirus (PHEV) and dromedary camel coronavirus (Dc-CoV-HKU23) do interpolate into the BCoV clade when their spike, nucleocapsid and membrane protein sequences are included. Therefore, we concede that the question of whether or not recombination within the genus, prior to, or after, the divergence of BCoV and HCoV-OC43, renders the phylogenetic approach invalid, must remain open. Only a more detailed knowledge of other viruses within the genus *Betacoronavirus*, and a more detailed study of their intra-specific and inter-specific recombination signals can provide the necessary reassurance. This should be a priority for future work on the origins of these coronaviruses.

In this respect, it should be noted that our sets S&G-2022-O and S&G-2022-W, which represent the identified non-recombinant region of Forni et al. [20] and the entire genome, respectively, produce mean MRCA values that are about 15 to 20 years apart. It should also be noted that the mean coefficientOfVariation parameter for S&G-2022-W strays into an area where it would be justifiable to reject the dataset on the grounds of too much branch rate heterogeneity. This can only be saved by the replacement of the coalescent constant size tree model with a Bayesian skyline. Together, these two issues support the suggestion made by Otieno et al. [21] that recombination is problematic. This then calls into question the use of those genes outside of the non-recombinant region identified by Forni et al. [20]. Based on such a stringent requirement, only dataset S&G-2022-O is likely to provide a suitable starting point to address the question. If that is the case, then we must conclude that the likeliest date for the emergence of HCoV-OC43 from BCoV is 1898 to 1902, based on the three mean MRCAs from the three substitution models applied, with the boundaries of their 95% HPD ranges between 1835 and 1953. This is a decade too late for RICT, but does permit us to speculate about alternative scenarios.

Does this new estimate of HCoV-OC43 emergence around 1898 to 1902 match with candidate pandemic events in the historical record? Table 7 shows that there was indeed another excess deaths spike in England and Wales, in 1899–1900 inclusive. At 1576 dpm excess, this is less than both the “Russian influenza” and COVID-19 (for 2020 and 2021 inclusive). This was not attributed to influenza at the time, with only a “minor prevalence” of influenza being recorded in March 1899 [3]. Despite the advantage that Parsons’ eyewitness perspective provides, subsequent authors have nevertheless inferred that there was some kind of pandemic event at that time. Worobey et al. [23] refer to a “putative pandemic around 1900” which is “retrospectively identified on the basis of increased pneumonia and influenza mortality in North America, England, Ireland, and elsewhere but is not universally considered a *bona fide* pandemic”. The retrospective identification these authors refer to appears to come, via a reference trail through several papers, ultimately from Collins in 1957 [27] who identifies Spring 1900 in Massachusetts as a time of excess deaths from “influenza and pneumonia”.

**Table 7:** Excess mortality for England & Wales calculated for years 1899 to 1900.

Year	Deaths per million (dpm) *per annum* (*p*.*a*)	Excess dpm *p*.*a*. vs. 1894–1898	1899–1900 total excess dpm
**1900**	18228	778	1576
**1899**	18249	799	
**1898**	17518		
**1897**	17379		
**1896**	17100		
**1895**	18685		
**1894**	16570		

Excess dpm *p*.*a*. are calculated against the average dpm *p*.*a*. of the 5 previous years 1894–1898. Source: kindly provided on email request from Office for National Statistics, UK.

Search of British newspapers for references to pneumonia in 1900 is complicated by the large number of pneumonia deaths reported for soldiers serving in the Boer War in South Africa. Nevertheless, *The Times* on 4^th^ January 1900 reports that “New Year finds us once more in the throes of Influenza, and if, happily, the attacks are not so severe as in some previous years, there can be no doubt that the epidemic is more widely spread than on any former occasion. A glance at the mortality returns shows, too, that in many quarters the outbreak is a particularly serious one.” On the 23^rd^ January 1900, we are told that “the scythe of death has swept unsparingly at home among high and low. The influenza epidemic has produced a widely-spread and deep-seated feeling of depression, which has reacted fatally on the vital forces of the aged and the infirm.” It was not until 10^th^ March 1900, that *The Times* was able to report that “the influenza epidemic is abating”. Perhaps the epidemic of winter 1899 to 1900, rather than the “Russian influenza” pandemic of 1890, provides the best candidate for the emergence of HCoV-OC43 into humans.

In summary, we reviewed all publications which use molecular data pertinent to the hypothesis we term RICT, downloading their provided datasets or reconstructing them from descriptions given in those articles. To this group of datasets, we added our own. Preliminary assessment led us to discard certain datasets as unsuitable for Bayesian analysis. For the remainder, we derive mean MRCA values that are all too late to fit RICT, with the exception of one early dataset (V-2006-S). While recognising that the confidence limits of these MRCA dates still overlap with the dates of the “Russian influenza” pandemic, we conclude that the weight of molecular evidence from several independent studies, as well as our own presented here, argues against RICT. Human coronavirus OC43 is more likely to have emerged from bovine coronavirus at a date after the “Russian influenza” pandemic. We also present historical evidence that suggests a tentative date of 1899–1900 for the emergence of OC43.
